# Fibroblast Growth Factor 9 Activates Akt and MAPK Pathways to Stimulate Steroidogenesis in Mouse Leydig Cells

**DOI:** 10.1371/journal.pone.0090243

**Published:** 2014-03-06

**Authors:** Meng-Shao Lai, Yu-Sheng Cheng, Pei-Rong Chen, Shaw-Jenq Tsai, Bu-Miin Huang

**Affiliations:** 1 Institute of Basic Medicine, College of Medicine, National Cheng Kung University, Tainan, Taiwan, Republic of China; 2 Department of Cell Biology and Anatomy, College of Medicine, National Cheng Kung University, Tainan, Taiwan, Republic of China; 3 Department of Urology, National Cheng Kung University Hospital Douliou Branch, Yunlin, Taiwan, Republic of China; 4 Department of Physiology, College of Medicine, National Cheng Kung University, Tainan, Taiwan, Republic of China; Taipei Medical University, Taiwan

## Abstract

Fibroblast growth factor 9 (FGF9) is a multifunctional polypeptide belonging to the FGF family and has functions related to bone formation, lens-fiber differentiation, nerve development, gap-junction formation and sex determination. In a previous study, we demonstrated that FGF9 stimulates the production of testosterone in mouse Leydig cells. In the present study, we used both primary mouse Leydig cells and MA-10 mouse Leydig tumor cells to further investigate the molecular mechanism of FGF9-stimulated steroidogenesis. Results showed that FGF9 significantly activated steroidogenesis in both mouse primary and tumor Leydig cells (*p*<0.05). Furthermore, FGF9 significantly induced the expression of phospho-Akt at 0.5 and 24 hr, phospho-JNK at 0.25, 0.5, and 24 hr, phospho-p38 at 0.5 hr, and phospho-ERK1/2 from 0.25 to 24 hr in primary Leydig cells (*p*<0.05). Also, FGF9 significantly up-regulated the expression of phospho-Akt at 3 hr, phospho-JNK at 0.25 hr, and phospho-ERK1/2 at 1 and 3 hr in MA-10 cells (*p*<0.05). Using specific inhibitors of Akt, JNK, p38, and ERK1/2, we further demonstrated that the inhibitors of Akt and ERK1/2 significantly suppressed the stimulatory effect of FGF9 on steroidogenesis in mouse Leydig cells. In conclusion, FGF9 specifically activated the Akt and ERK1/2 in normal mouse Leydig cells and the Akt, JNK and ERK1/2 in MA-10 mouse Leydig tumor cells to stimulate steroidogenesis.

## Introduction

Fibroblast growth factor 9 (FGF9) was first isolated from the culture supernatant of the human glioma cell line NMC-G1 [1] and has been shown to participate in neuron development, bone formation, lens-fiber differentiation, gap-junction formation, sex determination, and steroidogenesis [2]–[8]. In fact, studies have demonstrated that FGF9 is expressed in the embryonic and postnatal male reproductive tract [3]. Moreover, FGF9-knockout mice die after birth as a consequence of hypoxia in lung cells and exhibit various phenomena, such as testicular hypoplasia and male-to-female sex reversion [3]. Other studies have shown that FGF9 participates in sex determination by maintaining SOX9 expression and seminiferous tubule formation, and failure of FGF9 expression results in abnormal testicular development in humans [9]–[11]. Together, these evidences suggest that FGF9 plays an important role in male gonadal development and function. Colvin's 2001 study showed that expression of the cholesterol side chain cleavage enzyme (Scc) decreases in FGF9-knockout mice [3]. In a previous study, we demonstrated that FGF9 increases testosterone production in postnatal mouse Leydig cells [8], implying that FGF9 may regulate steroidogenesis in male reproductive tissues.

Testosterone promotes gonad development and maintains reproductive functions in male adults [12]. Furthermore, steroidogenesis in Leydig cells is regulated by luteinizing hormone/chorionic gonadotropin (LH/CG), and binding of LH to its receptor on the surface of Leydig cells triggers the Gs/adenylyl cyclase/cAMP/PKA signaling pathways [13]. Although PKA-mediated protein phosphorylation is undoubtedly important in regulating steroidogenesis, other signaling systems have also been implicated, including protein kinase C (PKC) [14], mitogen-activated protein kinase (MAPK) [15], and phosphoinositide 3-kinase (PI3K) [16].

FGF receptor (FGFR) is a member of the receptor tyrosine kinase family and plays important roles in cell proliferation and survival under diverse stimulation of growth factors [17], [18]. Binding of FGF to FGFR initiates various signal transduction pathways, such as the MAPK [19], PI3K [20], and phospholipase C gamma (PLCγ) pathways [21]. Additionally, MAPKs have three downstream signal relays to pass the growth factor signals, including the signals mediated by extracellular signal–regulated kinases 1 and 2 (Erk1/2), c-jun N-terminal kinase (JNK), and p38 kinase [22].

Our previous study demonstrated that FGF9 increases testosterone production principally through the Ras/MAPK and PI3K signaling pathways rather than the PLCγ pathway in Leydig cells [8]. In the present study, using both mouse primary and tumor Leydig cells, we further explored how FGF9 could activate JNK, p38, ERK1/2, and Akt, which are the downstream signal transduction mediators of the Ras/MAPK and PI3K pathways.

## Materials and Methods

### Chemicals

Human FGF9 was purchased from R&D Systems (Minneapolis, MN, USA). Scintillation fluid was purchased from PerkinElmer (Boston, MA, USA). Fetal bovine serum and M199 powder were purchased from Gibco (Grand Island, NY, USA). Nitroblue tetrazolium, β-nicotinamide adenine dinucleotide, dihydroepiandrosterone, EDTA, ethylene glycol tetraacetic acid, sodium pyrophosphate, β-glycerophosphate and sodium orthovanadate were purchased from Sigma (Seelze, Germany). Waymouth's MB752/1, PD98059, SP600125, SB203580, and wortmannin were purchased from Sigma (St. Louis, MO, USA). 1,2,6,7-3H(N)-testosterone (70.00 Ci/mmol) and 1,2,6,7-3H(N)-progesterone (70.00 Ci/mmol) in 0.25 ml ethanol were purchased from DuPont-New England Nuclear (Boston, MA, USA). Antibodies against phospho-ERK1/2, phospho-p38, phospho-JNK, and phospho-Akt were purchased from Cell Signaling (Beverly, MA, USA). Antibody against β-actin was purchased from Sigma (St. Louis, MO, USA).

### Animals

Male C57BL/6NCrj (B6) mice (5 to 6 weeks old) were purchased from Laboratory Animal Center of National Cheng Kung University, Tainan, Taiwan. All animals were housed under standard conditions in groups of four in 29×18×13 cm polyethylene cages at a temperature of 22–24°C and under a constant 12-hr light/dark cycle. Mice were provided with sterilized food and water ad libitum. We have followed the ARRIVE guidelines regarding animal studies [23].

### Ethics statement

All procedures for animal maintenance and sacrifice complied with the Guide for the Care and Use of Laboratory Animals and were approved by the Institutional Animal Care and Use Committee of National Cheng Kung University.

### Isolation of mouse primary Leydig cells

Leydig cells were isolated from 5- to 6-week-old immature B6 mice. Mouse testes were removed from sacrificed mice and decapsulated in M199 medium. Decapsulated testes were then incubated in a shaking water bath (120 cycles/min) at 37°C for 15 min in M199 containing 1% bovine serum albumin and 100 U/ml collagenase type II. Seminiferous tubules were separated from interstitial cells by gravity sedimentation. Cells were collected by centrifugation at 300× *g* for 6 min and resuspended in 2 ml of M199 containing 0.1% bovine serum albumin. This interstitial cell preparation was layered by a Percoll™ (GE Healthcare, Uppsala, Sweden) gradient and then centrifuged at 800× *g* at 4°C for 20 min. A 1-ml fraction of the gradient was collected from the top. Mouse Leydig cells were mainly distributed in fractions 23–25. 3β-HSD staining solution—which consisted of 1 mg/ml nitroblue tetrazolium, 3 mg/ml β-nicotinamide adenine dinucleotide, 2.88 mg/ml dihydroepiandrosterone in 10% acetone, and 1.6 mg/ml nicotinamide in 0.07 M phosphoric acid solution at pH 7.4—was used to identify the purity of Leydig cells. After isolation, cells were treated with 3β-HSD staining solution at 37°C for 1 hr [8]. 3β-HSD-positive cells represent the Leydig cells, and all our preparations reached 85–95% purity.

### Cell culture and treatments

MA-10 mouse Leydig tumor cells were generously provided by Dr. Mario Ascoli (University of Iowa, Iowa City, IA, USA), who originally developed and well characterized this cell clonal strain [24]. MA-10 cells were maintained in modified Waymouth's MB752/1 medium containing 24 mM HEPES, 1.12 g/l NaHCO_3_, and 0.04 g/l gentamicin sulfate and was supplemented with 10% fetal bovine serum. MA-10 cells (6×10^5^ cells/ml) were seeded in 6-cm dishes. After seeding for 20 hr, culture dishes were rinsed well to remove serum and non-seeded cells for the following experiments. In the co-treatment experiment with inhibitor in mouse primary Leydig cells, cells were pretreated with ERK inhibitor (PD98059, 25 µM), JNK inhibitor (SP600125, 1 µM), p38 inhibitor (SB202190, 1 µM), or Akt inhibitor (wortmannin, 100 nM) for 30 min and then incubated with FGF9 (50 ng/ml) for an additional 15 min. In the co-treatment experiment with inhibitor in MA-10 cells, cells were pretreated with ERK inhibitor (PD98059, 25 µM), JNK inhibitor (SP600125, 1 µM), or p38 inhibitor (SB202190, 1 µM) for 30 min or with Akt inhibitor (wortmannin, 100 nM) for 1 hr and then treated with FGF9 (50 ng/ml) for an additional 15 min.

### Radioimmunoassay (RIA)

Radioimmunoassay for the measurement of testosterone and progesterone levels was carried out as described [8], [25]. Media from cell cultures with different treatments were collected, and 25 µl of diluted sample was withdrawn and placed in a glass tube. Testosterone or progesterone antisera (100 µl) (gift from Dr. Paulus S Wang, National Yang Ming University, Taipei, Taiwan) and [^3^H]testosterone or [^3^H]progesterone were added, and the mixture was allowed to equilibrate at room temperature for 2 hr. Charcoal was added to each mixture, which was then incubated for 15 min at 4°C and then centrifuged for 10 min. The supernatant was poured into 2 ml of scintillation fluid, and samples were counted in a β-counter for 2 min.

### Immunoblotting

Protein (35 µg) was subjected to SDS-PAGE (12.5% acrylamide gels). The protein bands in the gel were transferred to a polyvinylidene difluoride membrane (0.45 micron pore size, PerkinElmer) at 400 mA for 1 hr. Each membrane was then incubated in blocking buffer (0.5% non-fat milk in 0.1% TBST) for 1 hr and then incubated in fresh blocking buffer containing the primary antibody for 16–18 hr at 4°C. After washing three times with 0.1% TBST, immunopositive bands were visualized by an enhanced chemiluminescence (ECL) detection kit using the EC3 BioImaging System (UVP, Upland, CA, USA). The optical density of each protein band was quantified by a Quantity One (PDI, Huntington Station, NY, USA) computer-assisted image analysis system. The amount of β-actin in each lane was determined as a loading control [25].

### Statistical analysis

Data are expressed as mean ± SEM of three separate experiments. One-way analysis of variance with the least significant difference test was used to assess the difference among groups or between control and treatment. The significant difference was represented as *p*<0.05.

## Results

### FGF9 increases steroidogenesis in both mouse primary and tumor Leydig cells

**Figure 1:**
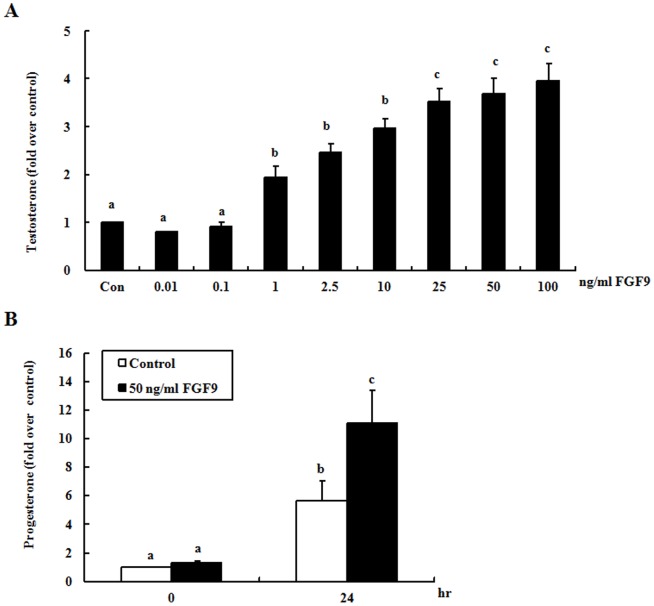
FGF9 increases steroidogenesis in both mouse primary and tumor Leydig cells. (A) Mouse primary Leydig cells were treated with different concentrations of FGF9 for 24 hr (Con = control). (B) MA-10 mouse Leydig tumor cells were treated without (control) or with 50 ng/ml FGF9 for 0 and 24 hr. At the end of each incubation, culture medium was collected, and testosterone (A) or progesterone (B) production was measured by RIA. Each bar represents the mean ± SEM of the fold difference in testosterone or progesterone production compared with the control group. Different letters above the bars indicate significant differences between treatments (*p*<0.05).

Mouse primary Leydig cells were treated with different concentrations of FGF9 (0, 0.01, 0.1, 1, 2.5, 10, 25, 50, and 100 ng/ml) for 24 hr. Results showed that FGF9 at induced testosterone production in a dose-dependent phenomenon. In fact, 1, 2.5, 10, 25, 50, and 100 ng/ml FGF9 stimulated 1.8-, 2.2-, 2.7-, 3.5-, 3.7-, and 4.0-fold increases of testosterone, respectively, compared with the control (Fig. 1A). Interestingly, the characteristics of cell death, such as rounded-up, membrane-blebbed and floating cells, were not observed in mouse primary Leydig cells treated with FGF9.

MA-10 cells are a mouse Leydig tumor cell line that produces progesterone rather than testosterone owing to a defect in 17α-hydroxylase [24]. In this study, MA-10 cells were treated with FGF9 (50 ng/ml) for 0 and 24 hr, and progesterone production was determined. After 24 hr incubation, progesterone concentration increased significantly from 8819 pg/ml (control) to 18,651 pg/ml (FGF9 treated), indicating 2.1-fold greater progesterone production in FGF9-treated cells (Fig. 1B). Similar to primary mouse Leydig cells, the characteristics of cell death were not observed in MA-10 cells with FGF9 treatments.

### Temporal effect of FGF9 on signaling through the Ras/MAPK and PI3K pathways in mouse primary Leydig cells

**Figure 2:**
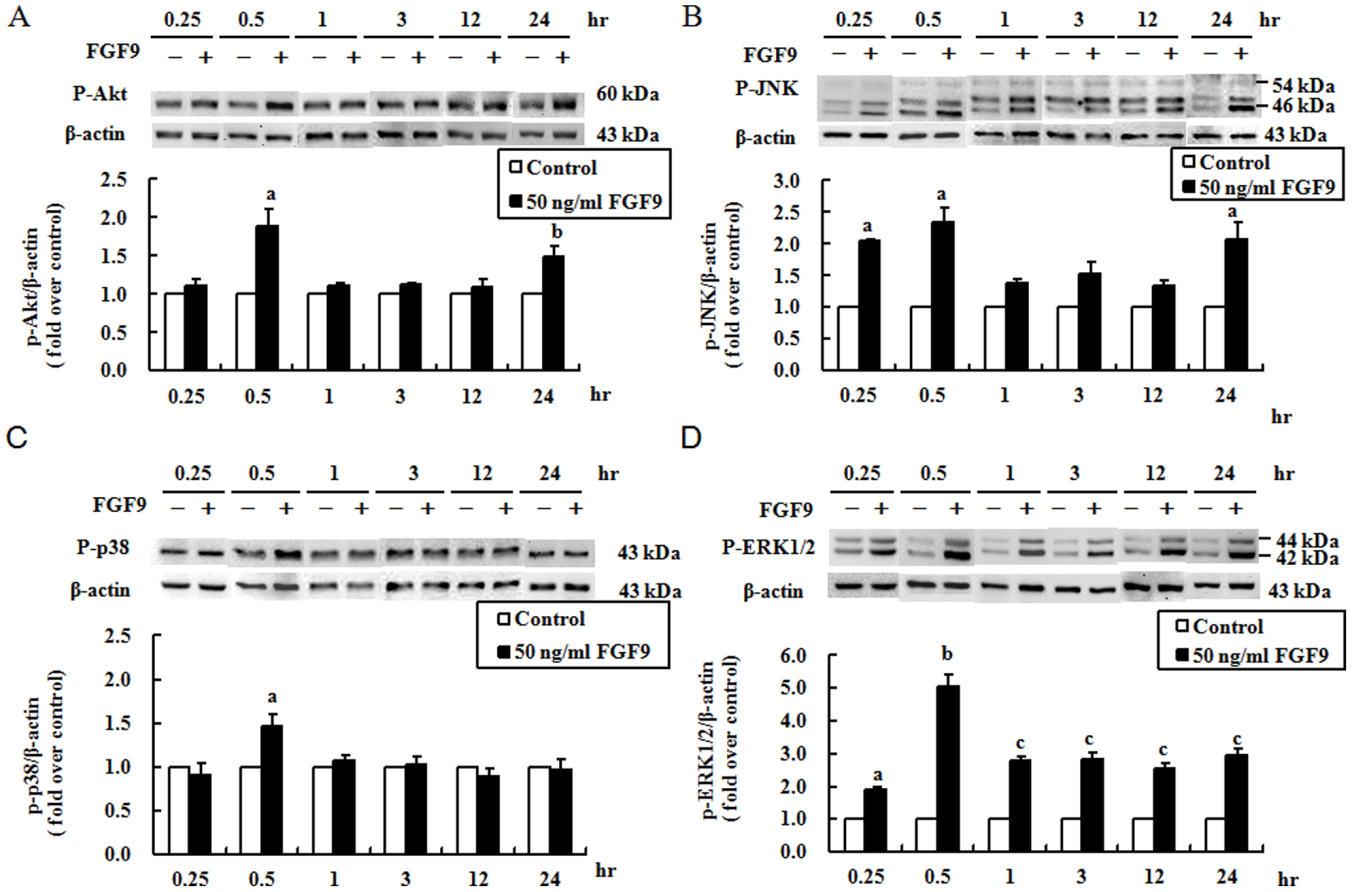
Temporal effect of FGF9 on the expression levels of phospho-Akt, -JNK, -p38, and -ERK1/2 in mouse primary Leydig cells. Mouse primary Leydig cells were treated without (control) or with FGF9 (50 ng/ml) for the indicated times. The expression levels of phospho-Akt (A), -JNK (B), -p38 (C), and -ERK1/2 (D) were detected by immunoblotting. Immunoblots represent the observations from one single experiment repeated in three different times. The integrated optical density of each protein was normalized to β-actin (43 kDa) in each lane. The fold difference of each of phospho-Akt, -JNK, -p38, and -ERK1/2 in the treatment group was normalized to the control group at each time point, and the mean ± SEM of three separate experiments is shown. Different letters above the bars indicate statistically significant differences between treatments (*p*<0.05).

Many studies have shown that Akt, JNK, p38, and ERK1/2 participate in steroidogenic signaling pathways [26]. In a previous study, we demonstrated that FGF9 activates the Ras/MAPK and PI3K pathways to increase steroidogenesis in primary mouse Leydig cells [8]. Here, we further clarified the downstream signals along these two pathways after FGF9 treatment in primary mouse Leydig cells by profiling the activation of Akt, JNK, p38, and ERK1/2 at 0.25, 0.5, 1, 3, 12, and 24 hr post-treatment. FGF9 significantly stimulated the expression of phospho-Akt at 0.5 and 24 hr (*p*<0.05; Fig. 2A), the expression of phospho-JNK at 0.25, 0.5, and 24 hr (*p*<0.05; Fig. 2B), the expression of phospho-p38 at 0.5 hr (*p*<0.05; Fig. 2C), and the expression of phospho-ERK1/2 at all time points (*p*<0.05; Fig. 2D).

### Temporal effect of FGF9 on signaling through the Ras/MAPK and PI3K pathways in MA-10 mouse Leydig tumor cells

**Figure 3:**
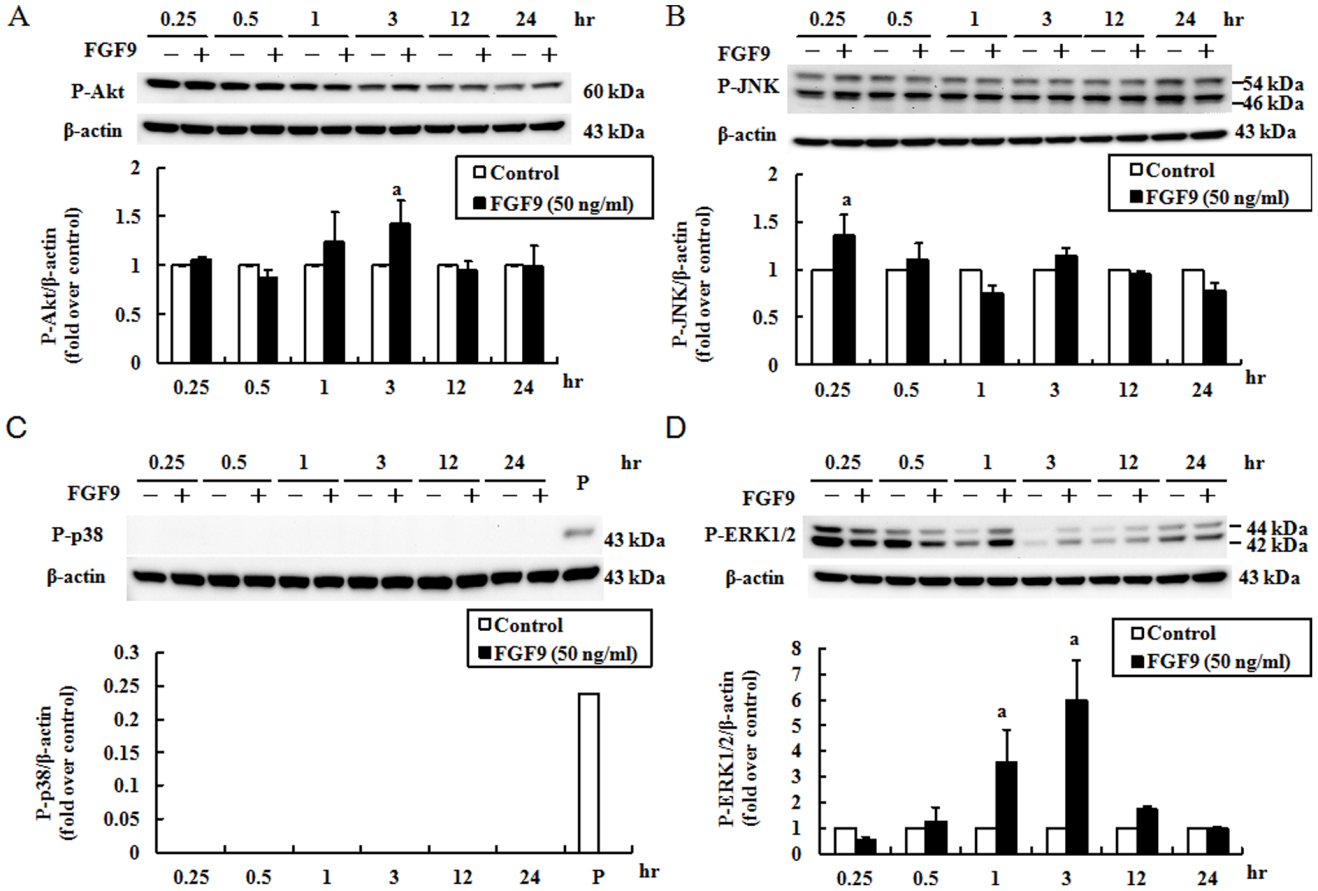
Temporal effect of FGF9 on the expression levels of phospho-Akt, -JNK, -p38, and -ERK1/2 in MA-10 cells. MA-10 cells were treated without or with FGF9 (50 ng/ml) for the indicated times. The expression levels of phospho- Akt (A), -JNK (B), -p38 (C), and -ERK1/2 (D) were detected by immunoblotting. Immunoblots represent the observations from one single experiment repeated at least in three different times. The integrated optical density of each protein was normalized to β-actin (43 kDa) in each lane. The fold difference of each of phospho-Akt, -JNK, -p38, and -ERK1/2 in the treatment group was normalized to the control group at each time point, and the mean ± SEM of three separate experiments is shown. Different letters above the bars indicate statistically significant differences between treatments (*p*<0.05). P represents the positive control.

We also used MA-10 cells to determine the activation profiles of Akt, JNK, p38 and ERK1/2 regulated by FGF9 after 0.25, 0.5, 1, 3, 12, and 24 hr of treatment. As shown in Figure 3, FGF9 significantly stimulated the expression of phospho-Akt at 3 hr (*p*<0.05; Fig. 3A), the expression of phospho-JNK at 0.25 hr (*p*<0.05; Fig. 3B), the expression of phospho-ERK1/2 at 1 and 3 hr (*p*<0.05; Fig. 3D), but not expression of phospho-p38 (*p*<0.05; Fig. 3C).

### Effect of inhibitors on FGF9-activated Akt, JNK, p38, and ERK1/2 phosphorylation in mouse primary Leydig cells

**Figure 4:**
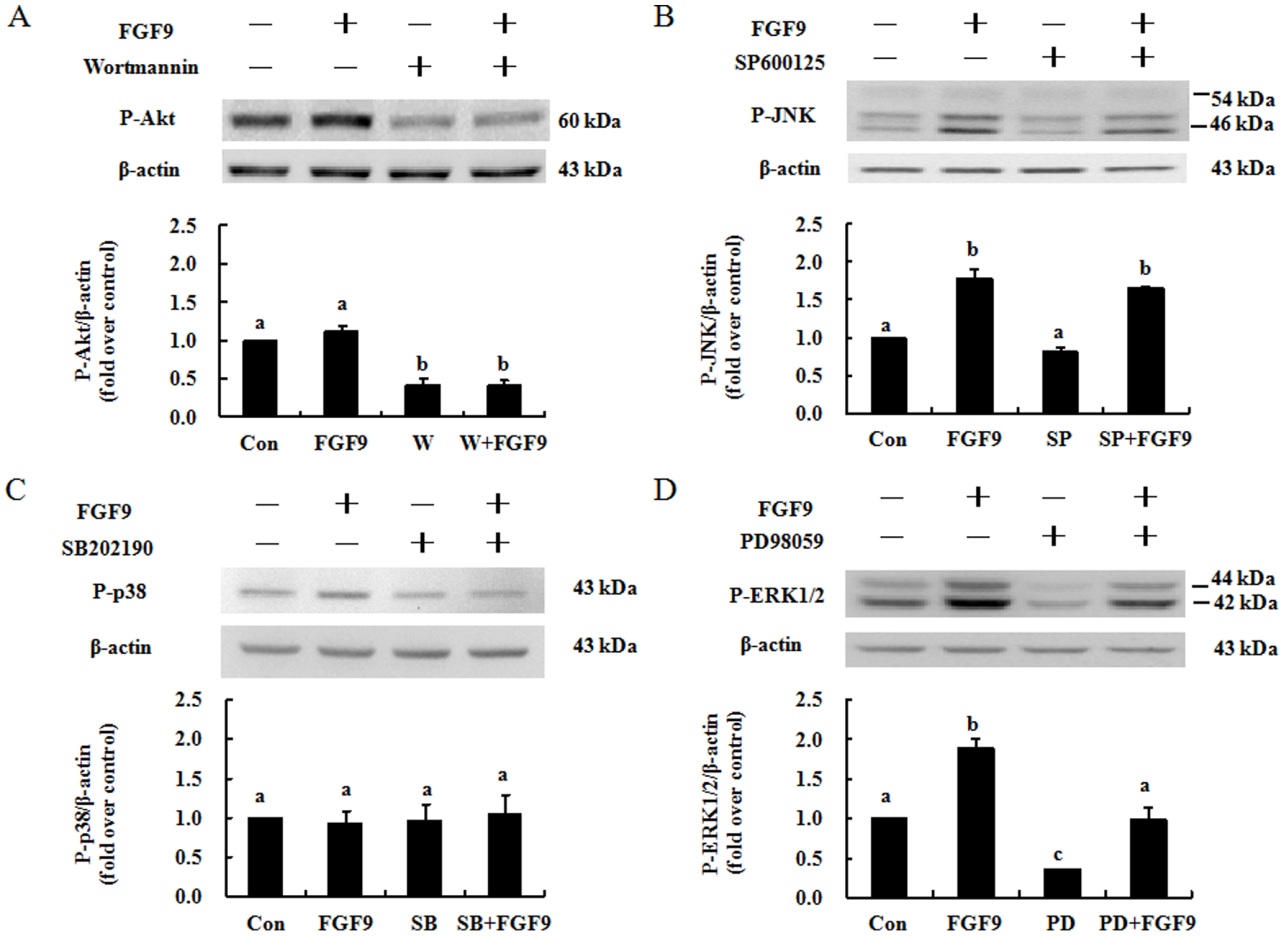
Effect of inhibitors on FGF9-induced activation of Akt, JNK, p38, and ERK1/2 in mouse primary Leydig cells. Mouse primary Leydig cells were pretreated with the Akt inhibitor wortmannin (100 nM) (A), JNK inhibitor SP600125 (1 µM) (B), p38 inhibitor SB202190 (1 µM) (C), or ERK inhibitor PD98059 (25 µM) (D) for 30 min and then incubated without or with FGF9 (50 ng/ml) for an additional 15 min. Immunoblots represent the observations from one single experiment repeated at least in three different times. The integrated optical density of each protein was normalized by β-actin (43 kDa) in each lane. Each bar represents the mean ± SEM fold difference of phospho-Akt, -JNK, -p38, and -ERK1/2 compared with the control group (Con) from three independent experiments. Different letters above the bars indicate statistically significant differences between treatments (*p*<0.05). W, wortmannin; SP, SP600125; SB, SB202190; PD, PD98059.

To determine whether FGF9 induces Akt, JNK, p38, and/or ERK1/2 phosphorylation, mouse primary Leydig cells were treated with specific inhibitors. Data showed that the Akt inhibitor wortmannin (100 nM) reduced phosphorylation of Akt in control and FGF9-treated cells (Fig. 4A), the JNK inhibitor SP600125 (1 µM) had no effect on phosphorylation of JNK in control or treated cells (Fig. 4B), the p38 inhibitor SB202190 (1 µM) had no effect on phosphorylation of p38 in control or treated cells (Fig. 4C), and the ERK1/2 inhibitor PD98059 (25 µM) reduced phosphorylation of ERK1/2 in control and treated cells (Fig. 4D).

### Effect of inhibitors on FGF9-activated Akt, JNK, p38, and ERK1/2 phosphorylation in MA-10 mouse Leydig tumor cells

**Figure 5:**
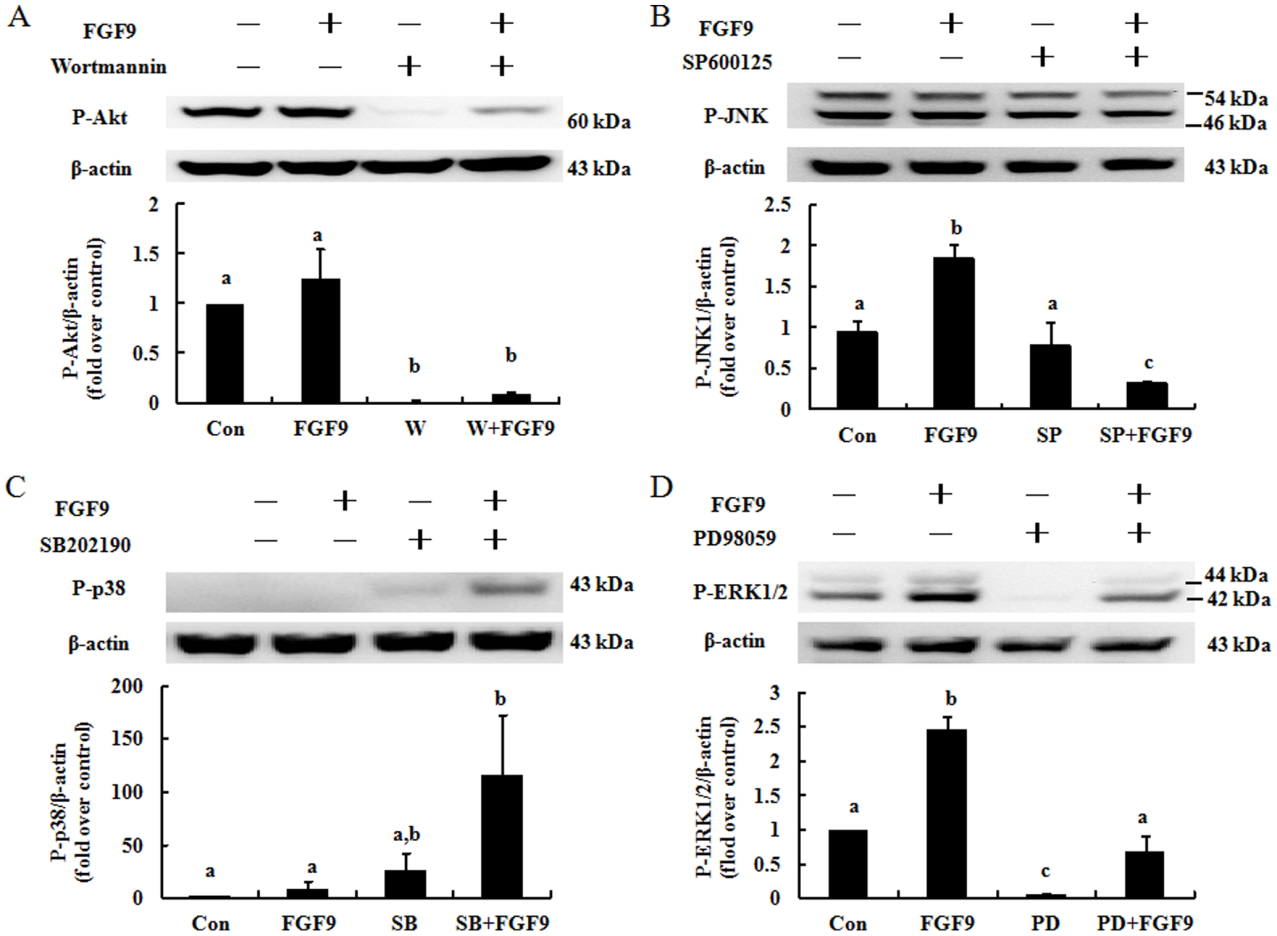
Effect of inhibitors on FGF9-induced activation of Akt, JNK, p38, and ERK1/2 in MA-10 cells. MA-10 cells were pretreated with the Akt inhibitor wortmannin (100 nM) for 1 hr (A), JNK inhibitor SP600125 (100 µM) for 30 min (B), p38 inhibitor SB202190 (1 µM) for 30 min (C), or ERK inhibitor PD98059 (25 µM) for 30 min (D). Then, cells were incubated without or with FGF9 (50 ng/ml) for 3 hr in the Akt inhibitor group, or for 15 min in the JNK, p38, and ERK1/2 inhibitor groups. Immunoblots represent the observations from one single experiment repeated at least in three different times. The integrated optical density of each protein was normalized by β–actin (43 kDa) in each lane. Each bar represents the mean ± SEM fold difference of phospho-Akt, -JNK, -p38, and -ERK1/2 compared with the control group (Con) from three independent experiments. Different letters above the bars indicate statistically significant differences between treatments (*p*<0.05). W, wortmannin; SP, SP600125; SB, SB202190; PD, PD98059.

In MA-10 mouse Leydig tumor cells, the Akt inhibitor wortmannin (100 nM) reduced phosphorylation of Akt in control and FGF9-treated cells (Fig. 5A), the JNK inhibitor SP600125 (100 µM) reduced phosphorylation of JNK in treated cells (Fig. 5B), the p38 inhibitor SB202190 (1 µM) induced phosphorylation of p38 in treated cells (Fig. 5C), and the ERK1/2 inhibitor PD98059 (25 µM) reduced phosphorylation of ERK1/2 in control and treated cells (Fig. 5D).

## Discussion

Previously, we demonstrated that FGF9 increases testosterone production in mouse primary Leydig cells through the Ras/MAPK, PI3K, and PKA pathways but not through the PLCγ pathway [8]. In the present study, we further showed that FGF9 could induce testosterone and progesterone production in mouse primary and tumor Leydig cells, respectively, through the induction of phospho-Akt, -JNK, -p38, and/or -ERK1/2, which are the downstream components of Ras and MAPK signal transduction pathways.

Studies have shown that the binding of FGF to FGFR initiates the PI3K pathway [20], and steroidogenesis is regulated by the PI3K pathway in Leydig cells [16]. Moreover, factors such prohibitin and restistin can activate the PI3K/Akt signaling pathway to regulate steroidogenesis in bovine and rat granulosa cells [27], [28]. In the present study, we showed that FGF9 activated the phosphorylation of Akt both in mouse primary and tumor Leydig cells, which is consistent with those studies.

Studies have demonstrated that steroidogenesis is regulated by many signaling pathways, such as the cAMP/PKA, PI3K, and MAPK pathways [13], [26]. It has been shown that gonadotropin releasing hormone (GnRH) activates ERK1/2, rather than JNK or p38, to increase transcription/translation of the gene encoding 3β-HSD and subsequent testosterone production in rat primary Leydig cells [29]. Furthermore, 8-Br-cAMP induces transcription/translation of steroidogenic acute regulatory (StAR) protein and progesterone production through ERK1/2 activation in MA-10 Leydig cells [15]. Moreover, bisphenol A, an endocrine disrupter, activates the CRE, PKA, Akt, and MAP kinase signaling pathways to regulate testosterone production in rat Leydig cells [30]. Thus, there is no doubt that JNK, p38, and ERK1/2 may be involved in regulating steroidogenesis. Parallel to those studies, we showed that FGF9 also activated the phosphorylation of JNK, ERK1/2, and/or p38 in mouse primary and tumor Leydig cells.

In our results, a temporal difference in the phosphorylation of Akt, JNK, p38, and ERK1/2 between mouse primary and tumor Leydig cells was observed. In general, phospho-Akt, -JNK, -p38, and -ERK1/2 were induced by FGF9 within 30 min in mouse primary Leydig cells. However, the period prior to phosphorylation was more unpredictable in MA-10 mouse Leydig tumor cells in that FGF9 stimulated phospho-Akt at 3 hr, phospho-JNK at 15 min, phospho-ERK1/2 at 1 and 3 hr, but no expression of phospho-p38. Studies have clearly shown that normal and tumor cells may exhibit different protein expression patterns [31], [32]. Moreover, different expression of FGF9 and FGF9 receptors between normal and abnormal cells have been well documented [11], [33], [34]. Furthermore, studies have also shown that the binding of FGF to FGFR initiates various signal transduction pathways in different cell types [19]–[21]. Thus, our observations of differential temporal expression of phospho-Akt, -JNK, -p38, and -ERK1/2 between mouse primary and tumor Leydig cells is not unprecedented.

In the present study, p38 was activated by FGF9 in primary Leydig cells. However, FGF9 did not activate any phospho-p38 expression in MA-10 cells. Studies have shown that overexpression of oncogenic MAP 17 in breast tumors relies on p38 insensitivity to induce intracellular reactive oxygen species [35]. Furthermore, p38/MAPK activation participates in apoptosis in epithelial cells through an unknown cell elimination process [36]. Thus, the loss of p38 activation might be involved in mouse Leydig cell tumorigenesis, which should be further investigated.

Our data showed that inhibitors of the Akt and MAPK pathways (wortmannin, SP600125, SB202190, and PD98059) had different effects on mouse primary and tumor Leydig cells. Akt and ERK1/2 inhibitors suppressed the phosphorylation of Akt and ERK1/2 in the presence of FGF9 in both mouse primary and tumor Leydig cells. However, the JNK inhibitor (SP600125) had no effect on primary mouse Leydig cells but suppressed phosphorylation of JNK in MA-10 cells in the presence of FGF9. SP600125 has different apoptotic effects depending on differences in the level of galectin-7 between urothelial carcinomas and normal urothelium [37]. Thus, SP600125 may have different effects on mouse primary and tumor Leydig cells.

Interestingly, we found that the p38 inhibitor (SB202190) had no effect in mouse primary Leydig cells. However, SB202190 potentiated FGF9 to activate phospho-p38 expression in MA-10 cells. Studies have reported that SB202190 induces cell death by cross-inhibition of the PI3K-PKB/Akt-mTOR pathway, but not the p38 pathway [38], that SB202190- or SB203580-induced JNK activation is dependent on the MLK-3-MKK4/MKK7-dependent signal transduction pathway [39], and that SB202190 activates ERK1/2 to promote the progression of leukemia [40]. These reports suggest that SB202190 may regulate other signaling pathways among various cell types rather than inhibit p38 activity. Thus, SB202190 and FGF9 may mutually potentiate p38 phosphorylation in MA-10 cells, which should be further investigated].

In conclusion, FGF9 specifically activated Akt and ERK1/2 in mouse primary Leydig cells and activated Akt, JNK, and ERK1/2 in MA-10 mouse Leydig tumor cells to stimulate steroidogenesis. Our study not only illustrates the relationship between FGF9 and Leydig cell functions but also reveals different properties between normal and tumor Leydig cells.
